# Soluble platelet-endothelial cell adhesion molecule-1, a biomarker of ventilator-induced lung injury

**DOI:** 10.1186/cc13754

**Published:** 2014-03-03

**Authors:** Jesús Villar, Mercedes Muros, Nuria E Cabrera-Benítez, Francisco Valladares, Milagros López-Hernández, Carlos Flores, José L Martín-Barrasa, Jesús Blanco, Mingyao Liu, Robert M Kacmarek

**Affiliations:** 1 CIBER de Enfermedades Respiratorias, Instituto de Salud Carlos III, Madrid, Spain; 2 Multidisciplinary Organ Dysfunction Evaluation Research Network, Research Unit, Hospital Universitario Dr. Negrin, Las Palmas de Gran Canaria, Spain; 3 Keenan Research Center for Biomedical Science at the Li Ka Shing Knowledge Institute, St. Michael’s Hospital, Toronto, Canada; 4 Department of Clinical Biochemistry, Hospital Universitario NS de Candelaria, Tenerife, Spain; 5 Department of Anatomy, Pathology & Histology, University of La Laguna, Tenerife, Spain; 6 Research Unit, Hospital Universitario N.S. de Candelaria, Santa Cruz de Tenerife, Spain; 7 Intensive Care Unit, Hospital Universitario Río Hortega, Valladolid, Spain; 8 Institute of Medical Science, Faculty of Medicine, University of Toronto, Toronto, Canada; 9 Respiratory and Critical Care Research Group, Toronto General Research Institute, University Health Network, Toronto, Canada; 10 Department of Respiratory Care, Massachusetts General Hospital, Boston, Massachusetts, USA; 11 Department of Anesthesiology, Harvard University, Boston, Massachusetts, USA

## Abstract

**Introduction:**

Endothelial cell injury is an important component of acute lung injury. Platelet-endothelial cell adhesion molecule-1 (PECAM1) is a transmembrane protein that connects endothelial cells to one another and can be detected as a soluble, truncated protein (sPECAM1) in serum. We hypothesized that injurious mechanical ventilation (MV) leads to shedding of PECAM1 from lung endothelial cells resulting in increasing sPECAM1 levels in the systemic circulation.

**Methods:**

We studied 36 Sprague–Dawley rats in two prospective, randomized, controlled studies (healthy and septic) using established animal models of ventilator-induced lung injury. Animals (*n* = 6 in each group) were randomized to spontaneous breathing or two MV strategies: low tidal volume (V_T_) (6 ml/kg) and high-V_T_ (20 ml/kg) on 2 cmH_2_O of positive end-expiratory pressure (PEEP). In low-V_T_ septic animals, 10 cmH_2_O of PEEP was applied. We performed pulmonary histological and physiological evaluation and measured lung PECAM1 protein content and serum sPECAM1 levels after four hours ventilation period.

**Results:**

High-V_T_ MV caused severe lung injury in healthy and septic animals, and decreased lung PECAM1 protein content (*P* < 0.001). Animals on high-V_T_ had a four- to six-fold increase of mean sPECAM1 serum levels than the unventilated counterpart (35.4 ± 10.4 versus 5.6 ± 1.7 ng/ml in healthy rats; 156.8 ± 47.6 versus 35.6 ± 12.6 ng/ml in septic rats) (*P* < 0.0001). Low-V_T_ MV prevented these changes. Levels of sPECAM1 in healthy animals on high-V_T_ MV paralleled the sPECAM1 levels of non-ventilated septic animals.

**Conclusions:**

Our findings suggest that circulating sPECAM1 may represent a promising biomarker for the detection and monitoring of ventilator-induced lung injury.

## Introduction

Mechanical ventilation (MV) is the most important supportive tool in the care of critically ill patients with acute respiratory failure. However, although necessary to preserve life, MV can itself aggravate or cause lung damage through a variety of mechanisms collectively referred to as ventilator-induced lung injury (VILI) [1]. These mechanisms include exposure to high inflation pressures, alveolar overdistension, and repetitive opening and closing of alveoli. In addition to direct structural damage to the lungs, these mechanical forces may lead to up-regulated cytokine release and a systemic inflammatory response, propagating injury to non-pulmonary organs, which may result in multiple organ failure, and ultimately affect outcome [2-4]. Our improved understanding of acute lung injury and VILI in patients with the acute respiratory distress syndrome (ARDS) has been important in designing lung-protective MV strategies aimed at attenuating VILI and improving outcomes. The only strategy that has demonstrated improved survival in ARDS patients is the use of low tidal volume (V_T_) MV with adequate positive end-expiratory pressure (PEEP) and limited transpulmonary distending pressure [5]. This strategy aims to minimize VILI which may result from alveolar overdistension or repeated opening and closing of lung units.

Considerable effort has been made to enhance our mechanistic understanding of VILI. Endothelial cell injury is an important component of ARDS and VILI, and the most fundamental early physiologic characteristic is an increase in protein permeability across the endothelial barrier of the lung. The damage observed in ARDS and VILI reflects the primary injurious stimuli and the secondary complex interactions of inflammatory mediators on alveolar epithelial and capillary endothelial cells [1,4]. Mechanical forces within the alveolus can cause endothelial disruption and hemorrhagic injury, even in the absence of pre-existing inflammation [6], and lead to activation of downstream messenger systems [7-11]. Platelet-endothelial cell adhesion molecule-1 (PECAM1) is a 130 kDa transmembrane protein member of the immunoglobulin superfamily of cell adhesion molecules that is constitutively localized at cell-cell junctions that connect endothelial cells to one another [12]. PECAM1 can be cleaved from endothelial cells, resulting in a secreted, shed protein (sPECAM1) [13,14]. Since pulmonary inflammation is a central component of VILI, and PECAM1 has been shown to mediate endothelial cell permeability [12], we hypothesized that PECAM1 can be cleaved and shed from the surface of endothelial cells and detected in the systemic circulation after a short term of injurious MV in an experimental, clinically relevant animal model of VILI using healthy and septic animals.

## Materials and methods

All experimental protocols were approved by the Animal Use and Care Committees at Hospital Universitario Dr. Negrin (Las Palmas, Spain) and Hospital Universitario NS de Candelaria (Tenerife, Spain) in accordance with the European Commission Directive 2010/63/EU for animal experimentation.

### Animal preparation and experimental protocol

We studied 36 male healthy and septic Sprague–Dawley rats weighing 300 to 350 g included in two prospective, randomized, controlled studies that survived after a four-hour ventilation period using two well-established models of VILI. Briefly, in the first animal model of VILI, anesthetized (intraperitoneal injection of 80 mg/kg ketamine hydrochloride and 8 mg/kg xylazine) healthy animals were randomized into three groups (n = 6 in each group): (1) spontaneous breathing; (2) MV with low-V_T_ (6 ml/kg) plus 2 cmH_2_O of PEEP; and (3) MV high-V_T_ (20 ml/kg) plus 2 cmH_2_O of PEEP. In the animal model of sepsis-induced lung injury, sepsis was induced by cecal ligation and puncture (CLP). A detailed description of this experimental model is provided elsewhere [10]. Eighteen surviving septic animals at 18 hours after CLP were anesthetized and randomly assigned to three groups (n = 6 in each group): (4) spontaneous breathing; (5) MV with low-V_T_ plus 10 cmH_2_O of PEEP; and (6) MV with high-V_T_ plus 2 cmH_2_O of PEEP. In healthy and septic animals assigned to MV, we performed a tracheotomy using a 14-G Teflon catheter. Thereafter, animals were paralyzed with 1 mg/kg pancuronium bromide and connected to a time-cycled, volume-limited rodent ventilator (Ugo Basile, Varese, Italy). During MV, healthy animals were on fraction of inspired oxygen (FiO_2_) = 0.4 and septic animals on FiO_2_ = 0.6. Respiratory rate was set to maintain constant minute ventilation in all ventilated groups.

The experimental settings were maintained for four hours, while animals were anesthetized and paralyzed by bolus administration of intraperitoneal ketamine/xylazine and pancuronium bromide every 45 minutes. Animals were monitored non-invasively to minimize the possibility of triggering an inflammatory response, after establishing a protocol which provided hemodynamic stability and comparable blood gases in invasively monitored animals during the four-hour experimental period. Peak airway pressures were continuously monitored. Oxygen saturation (SpO_2_) was continuously measured using a pulse oxymeter applied to the rat’s tongue. SpO_2_ remained ≥90% in all animals during animal instrumentation. Animals were maintained supine on a restraining board inclined 20° from the horizontal.

### Gas exchange and histological examination

At the end of the four-hour observation and ventilation period, a midline thoracotomy/laparotomy was performed and, after sampling arterial blood for gases and serum analysis by abdominal aorta puncture, animals were sacrificed by supplemental pentobarbital (10 mg/kg) and exsanguination after sectioning the abdominal vessels. The heart and lungs were removed ‘*en bloc*’. The lungs were isolated from the heart, the trachea was cannulated, and the right lung was fixed by intratracheal instillation of 3 ml 10% formalin. After fixation, lungs were floated in 10% formalin for a week. Then, lungs were sampled in multiple areas and sliced from apex to base and embedded in paraffin, then cut (3 μm thickness sections), stained with hematoxylin-eosin and examined by two pathologists (FV, MLH) who were blinded to group identity. Slides were viewed using a Nikon Optiphot light microscope (Tokyo, Japan) and photographed with a Nikon Digital DS-5 M camera (Tokyo, Japan) at × 200 magnification. Three random sections of the right lung from each animal were examined with particular reference to alveolar and interstitial damage defined as cellular inflammatory infiltrates, pulmonary edema, disorganization of lung parenchyma, alveolar rupture and hemorrhage. By scoring from 0 to 4 (none, mild, moderate, severe, very severe) for each parameter, a total histological injury score [15] was obtained by adding the individual scores in every animal and averaging the total values in each animal group.

### Protein extraction and PECAM1 immunoblotting

Left lungs from each experimental condition were excised, washed with saline, frozen in liquid nitrogen and stored at -80°C for subsequent protein extraction and blotting. Lungs were sampled in multiple areas, homogenized, and proteins were extracted by centrifugation (14,000 rpm) for five minutes at 4°C. Protein content in the supernatant of the extract was measured with Bio-Rad DC Protein Assay (Hercules, CA, USA). Western blotting was performed using a rabbit polyclonal anti-PECAM1 antibody (Santa Cruz Biotechnology, Santa Cruz, CA, USA). β-actin antibody (Cell Signaling Technology, Danvers, MA, USA) was used as loading control after stripping the membrane using Restore Western Blot Stripping Buffer. In all cases, bands were detected by chemiluminescence and measured by Scion Image software package (Scion Corp., Frederick, MD, USA).

### sPECAM1 serum levels

At the end of every experiment, blood collected in each animal was centrifuged for 15 minutes at 3,000 rpm. Sera were divided into aliquot portions and frozen at -80°C. sPECAM1 concentrations in serum were measured by enzyme-linked immunosorbent assay (ELISA) in dilutions that allowed interpolation from a simultaneously run standard curve. Levels of sPECAM1 were measured with a commercially available ELISA kit specific for rats (Cusabio, Wuhan Huamei Biotech, Wuhan, Hubei, China) following specifications of the manufacturer. Samples were analyzed using a fully automated ELISA analyzer (Triturus, Grifols, Spain). This assay has high sensitivity and excellent specificity for detection of rat sPECAM1 and eliminates interference by soluble receptors, binding proteins, and other factors present in biological samples. sPECAM1 concentrations are expressed as ng/ml. The detection range is 3.1 to 200 ng/ml.

### Data analysis

Data are expressed as mean ± standard deviation (SD). Comparisons involving all experimental groups were performed with one-way analysis of variance. We used a Bonferroni correction for multiple comparisons. Values derived from Western blot densitometry were expressed as group mean, normalized to β-actin and expressed as fold-changes of ventilated lungs versus control (non-ventilated) lungs, and tested with the same statistical analyses. Data analysis was performed using SPSS (SPSS Inc, Chicago, IL, USA). A two-sided *P*-value <0.05 was considered significant.

## Results

### Respiratory parameters and pathological evaluation

In healthy ventilated animals, mean peak inspiratory pressures were 14 ± 1 and 24 ± 2 cmH_2_O for the low-V_T_ and high-V_T_ groups, respectively (*P* <0.0001). In septic ventilated animals, mean peak inspiratory pressures were 20 ± 1 and 28 ± 2 cmH_2_O for the low-V_T_ and high-V_T_ groups, respectively (*P* <0.0001). Mean partial pressure of arterial CO_2_ (PaCO_2)_ did not differ among subgroups in each category (42 ± 2 versus 40 ± 2 mmHg for low-V_T_ versus high-V_T_ healthy groups; 42 ± 2 versus 39.5 ± 3 mmHg for low-V_T_ versus high-V_T_ septic groups).

**Figure 1:**
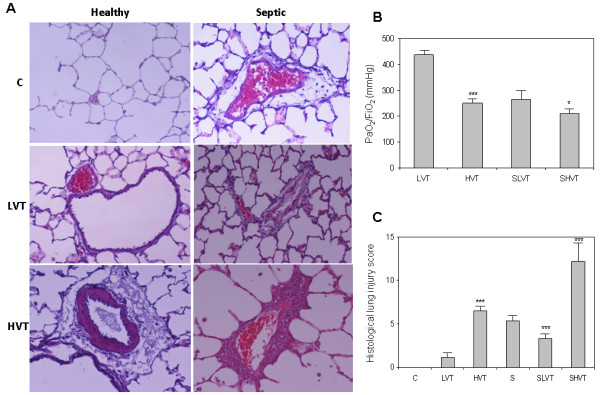
**Histological and gas-exchange findings. (A)** Representative histological features of different ventilatory strategies in healthy and septic lungs. Animals ventilated with high tidal volume showed abundant pulmonary infiltrates and perivascular edema (hematoxylin-eosin, ×200). **(B)** PaO_2_/FiO_2_ ratios at the end of a four-hour ventilation period. ***P <0.001 (HVT versus LVT); *P <0.05 (SHVT versus SLVT). **(C)** Histological lung injury score. ***P <0.001 (HVT versus LVT; SHVT versus S and SLVT; SLVT versus S). C: healthy control, spontaneous breathing lung; LVT: healthy, ventilated with low tidal volume; HVT: healthy lung ventilated with high tidal volume; S: septic, spontaneous breathing rats; SLVT: septic lung ventilated with low tidal volume; SHVT: septic lung ventilated with high tidal volume. PaO_2_/FiO_2,_ ratio of arterial partial pressure of oxygen to fraction of inspired oxygen.

Healthy animals ventilated with high-V_T_ and septic animals on spontaneous breathing or high-V_T_ MV had histological and gas exchange evidence of lung injury (Figure 1A, B). Mean ratio of arterial partial pressure of oxygen to fraction of inspired oxygen (PaO_2_/FiO_2_) in the low-V_T_ control group was over 400 mmHg; in contrast, mean PaO_2_/FiO_2_ in healthy high-V_T_ animals (250 ± 20 mmHg), and in septic animals ventilated with low-V_T_ plus PEEP (265 ± 38 mmHg) or with high-V_T_ (211 ± 28 mmHg) met oxygenation criteria for acute lung injury [5] (Figure 1B).

After four hours of high-V_T_ MV, the lungs showed acute inflammatory infiltrates and perivascular edema. Septic animals ventilated with high-V_T_ had the highest histological injury scores (12.2 ± 2.3) whereas healthy animals ventilated with low-V_T_ had the lowest score (1.1 ± 0.3) (Figure 1C). Septic animals ventilated with low-V_T_ plus PEEP had a lower histological score than septic, spontaneous breathing animals (3.3 ± 0.5 versus 5.4 ± 0.7) (*P* = 0.001). These results confirmed that high-V_T_ MV and sepsis alone caused acute lung injury, and high-V_T_ MV further enhanced sepsis-induced lung injury.

### Lung PECAM1 protein content

**Figure 2:**
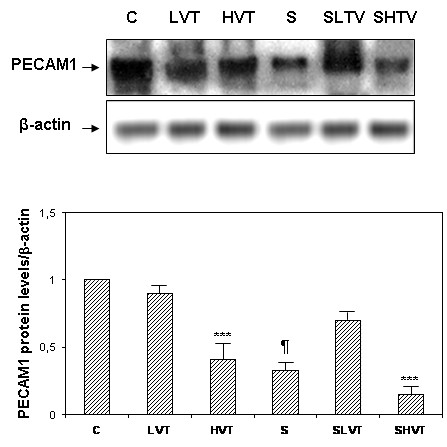
**Western blot and densitometry analysis of PECAM-1 protein in the lungs.** Experimental groups: healthy, spontaneous breathing control rats (C); healthy, ventilated with low tidal volume (LVT); healthy, ventilated with high tidal volume (HVT); septic, spontaneous breathing animals (S); septic, ventilated with low tidal volume (SLVT); septic, ventilated with high tidal volume (SHVT). ***P <0.001 (HVT versus C and LVT; SHVT versus S and SLVT); ¶ *P* <0.001 for S versus C. PECAM1, platelet endothelial cell adhesion molecule-1.

MV and sepsis decreased PECAM1 protein content in the lungs depending on the ventilatory strategy (Figure 2). Healthy animals from the non-ventilated and low-V_T_ groups had similar levels of PECAM1. However, high-V_T_ MV was accompanied by a marked reduction of PECAM1 lung levels (*P* <0.001). Overall, these data suggest an early reduction of cell-cell adhesions of endothelial cells in healthy and septic lungs after four hours of injurious MV.

### sPECAM1 serum levels

**Figure 3:**
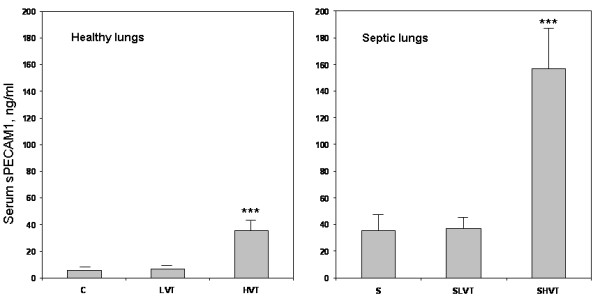
**Serum levels of soluble platelet endothelial cell adhesion molecule 1 (sPECAM1) in healthy and septic animals.** Experimental groups: healthy, spontaneous breathing control rats (C); healthy, ventilated with low tidal volume (LVT); healthy, ventilated with high tidal volume (HVT); septic, spontaneous breathing animals (S); septic, ventilated with low tidal volume (SLVT); septic, ventilated with high tidal volume (SHVT). ***P <0.0001 (HVT versus C and LVT; SHVT versus S and SLVT).

Animals on high-V_T_ MV had a four- to six-fold increase of mean sPECAM1 serum levels than their unventilated counterparts (35.4 ± 10.4 versus 5.6 ± 1.7 ng/ml in healthy rats; 156.8 ± 47.6 versus 35.6 ± 12.6 ng/ml in septic rats) (*P* <0.0001 for both comparisons) (Figure 3). However, low-V_T_ MV prevented such drastic changes when compared to the unventilated counterpart. Increases in the levels of sPECAM1 in healthy animals on high-V_T_ MV paralleled the increased levels of sPECAM1 of septic animals on spontaneous breathing and on low-V_T_ MV.

## Discussion

Our study suggests a new order of complexity in regulating the bridge between MV, sepsis and ARDS. The mechanisms of VILI have been extensively reviewed in the past 20 years [2,16,17]. Repeated overexpansion of the lung increases microvascular permeability and leads to edema formation without actual rupture of the lung. Electron microscopy has demonstrated ‘capillary stress fractures’ when microvascular pressures are markedly elevated [18]. It is this ‘stress failure’ of the alveolar capillary membrane that is responsible for the increased microvascular permeability edema seen with lung overinflation. Because the pulmonary endothelial barrier is damaged during sepsis, ARDS and VILI, lung cytokines released into the circulation can initiate or propagate a systemic inflammatory response and play an active role in the development of multiple system organ dysfunctions [19]. In our study, the application of low-V_T_ plus PEEP dramatically reduced lung injury in septic animals, a finding that has been previously reported by our group and by several other investigators [2,15,20,21].

The present study is the first to demonstrate that: (1) PECAM1 expression is modulated in the lungs in response to MV in the presence or absence of sepsis; (2) the expression of PECAM1 is dependent on the MV regime applied; and (3) the circulating levels of sPECAM1 are rapidly and markedly increased during injurious MV. PECAM1 is expressed on the surface of hematopoietic and immune cells and is highly enriched at endothelial cell-junctions [13,14,22]. Since its cloning in 1990 [23], there has been enormous progress in understanding the biology of PECAM1 and there is now clear evidence for its involvement in regulating leukocyte migration and inflammatory and vascular responses in numerous disease processes, including sepsis [12]. There is growing evidence that PECAM1 not only contributes to the maintenance of vascular integrity in resting cells but also to its restoration following barrier disruption [24] because deficiency in murine endothelial PECAM1 delays reestablishment of the vascular permeability barrier after histamine challenge [25] or in response to physical injury [26]. It has been reported that lung samples of patients who died from ARDS had a heterogeneous expression of PECAM1 in blood vessels [27]. Other studies have shown down-regulation of PECAM1 in neutrophils from patients with major trauma [28] and in the lungs of children who died from bronchopulmonary dysplasia after being mechanically ventilated [29]. Our data strongly suggest that the loss of PECAM1 in the lungs, which occurred in response to lung overstretch, compromises the permeability barrier and leads to edema formation and leukocyte infiltration (as seen by histological examination) and severe hypoxemia (as shown by a marked decrease of the PaO_2_/FiO_2_ ratio). Of note, lung PECAM1 content in septic animals ventilated with low-V_T_ plus 10 cmH_2_O of PEEP, a so-called protective MV strategy, remained higher, at levels very similar to non-septic animals. Also, the application of a protective MV strategy in animals with sepsis-induced lung injury prevented the increase of circulating serum levels of sPECAM1, which was associated with a lower histological lung injury score and a higher PECAM1 protein content in the lungs.

Little is known about the biochemical events involved in PECAM1 synthesis and processing in endothelial cells. Synthesis of PECAM1 is tightly regulated, as it is not found outside the vasculature. The specific mechanism by which PECAM1 functions to maintain or repair vascular barrier integrity is still poorly understood [24]. Levels of circulating PECAM1 particles shed from damaged endothelial cells have been reported as a marker of endothelial injury and to be present in various inflammatory diseases, including sepsis [14,30-34]. Current evidence suggests that activated inflammatory cells undergo an active shedding of the extracellular domain of the PECAM1 molecule which could contribute to the loss of cell-cell adhesion [13] and to the rise in circulating sPECAM1 during inflammatory diseases [14]. Our translational model of sepsis-induced lung injury is considered as the gold standard in sepsis and ARDS research that closely mimics the pathophysysiology of human sepsis [35] and ARDS [10]. In our study, the detection of elevated sPECAM1 serum levels in animals ventilated with high-V_T_ supports the concept of a primary endothelial cell involvement in the pathogenesis of increased vascular permeability seen in VILI and sepsis-induced ARDS. In this context, serial measurements of sPECAM1 levels could be used to assess prognosis or to monitor the benefits of endothelium-protective therapy. sPECAM1 is present in normal human plasma at levels of 10 to 25 ng/ml that are not associated with cellular debris or platelet microparticles [36]. Although the role of sPECAM1 serum levels *in vivo* remains to be determined, the membrane-anchored protein is required for transendothelial migration of leukocytes [12,37]. Thus, under normal conditions, the presence of low levels of sPECAM1 is indicative of normal functioning of PECAM1 as counteracting the tendency of leukocytes to leave the vasculature, whereas higher circulating levels of sPECAM1, as occurs during inflammatory conditions such as sepsis, ARDS and VILI, could serve as diagnostic and prognostic biomarkers of endothelial dysfunction.

Measuring circulating sPECAM1 levels could help to identify and monitor the management of septic and ARDS patients with different degrees of severity. Our findings on assessment of sPECAM1 levels under distinct modes of MV meet most of the criteria proposed by Shehabi and Seppelt [38] when seeking an ideal biomarker: ‘a SMART biomarker is Sensitive, Measurable (with a high degree of precision), Available (Affordable and safely Attainable), and Responsive (and Reproducible) in a Timely fashion to expedite clinical decision making’. A low sPECAM1 level is associated with normal or improved endothelial function and may be a marker of successful response to conventional therapy. By contrast, a high sPECAM1 level may be considered a marker of failure to respond to therapy and may require additional treatments to improve outcome.

We used a V_T_ of 20 ml/kg, a value that although higher than those used clinically, produces stretch that is likely comparable to that experienced by some patients with acute lung injury or ARDS in nondependent areas of the lung, even when relatively small V_T_ are used. In experimental models, VILI develops when a lung strain (estimated as the ratio between lung volume change and resting volume) greater than two is achieved, corresponding to V_T_ of approximately 20 ml/kg in healthy animals [39,40]. In our preliminary experiments, we found that 20 ml/kg was the minimal level of overdistension that caused an easily identifiable injury during a short period of MV. Supraphysiologic V_T_ has been used since the classical paper by Web and Tierney [20] for investigating lung injury in *in vivo* ventilated small experimental animals [41]. This approach reflects the compromise between limiting the ventilation period to a few hours (in order to limit the role of confounding factors such as anesthesia, fluid status, infection, nutrition and so on) and the attempt to produce detectable lung injury in a very short time. We used a level of 2 cmH_2_O of PEEP in animals with previously healthy lungs. This level of PEEP in healthy lungs is sufficient to avoid atelectasis during the four-hour MV period. There is no documented need for using high levels of PEEP during a short-term MV period in animals with healthy lungs. In a previous study by our group [6], we demonstrated that the use of 10 cmH_2_O to a constant large V_T_ (20 ml/kg) exacerbated VILI in previously healthy animals. However, in sepsis-induced lung injury animals, we used 10 cmH_2_O of PEEP in the low-V_T_ group since it is within the range of PEEP levels used in the management of patients with moderate ARDS. We used a FiO_2_ of 0.4 in healthy animals because in previous pilot studies, some animals ventilated with high-V_T_ and breathing room air developed hypoxemia at the end of the four-hour MV period. We used a FiO_2_ of 0.6 in animals with sepsis-induced lung injury because in previous experiments using 40% oxygen in the CLP model, some septic animals ventilated with high-V_T_ developed hypoxemia at the end of the four-hour MV period.

The present study has some limitations and strengths. First, we have not explored whether the application of protective MV after four hours of high-V_T_ MV could decrease the elevated sPECAM1 levels in healthy and septic animals with VILI. This should be determined in future experimental studies. Since PECAM1 is required for restoring endothelial continuity, targeting PECAM1 integrity may open new therapeutic approaches for sepsis and ARDS. In a model of endotoxic shock, Maas *et al*. [42] were able to transplant bone marrow cells expressing PECAM1 into PECAM1-deficient mice and found that the animals became resistant to endotoxic shock and maintained an intact vascular permeability barrier after endotoxin administration. Second, although both injurious MV and sepsis alone increased circulating sPECAM1 serum levels, our data demonstrated that sPECAM1 levels are more sensitive to MV than to the underlying disease process. This should be confirmed in future experimental and clinical studies. Third, pulmonary vascular stress was not assessed in our study and it may have contributed to the development of VILI [43-45]. However, based on our pilot data, the approaches used to ventilate these animals did not markedly increase intravascular stress.

Searching for biomarkers for sepsis, ARDS and VILI has been an on-going journey in critical care research. Many biomarkers proposed by experimental studies have not been able to be applied in clinical practice. The lack of convincing biomarkers further promotes investigations along this line. Usually, the experimental studies have a very well defined mechanical ventilation setting, inflammatory stimulus and other well-controlled conditions, while clinically we are dealing with patients in complicated situations. Therefore, it is critical to validate biomarkers clinically. It is also important to compare and combine different biomarkers in order to define and differentiate different clinical conditions. It is our hope that the present study will be able to stimulate more interest and discussion of sPECAM1 and other biomarkers for VILI.

## Conclusions

We have demonstrated that high-V_T_ MV decreased PECAM1 protein content in the lung and increased serum levels of sPECAM1. This effect was highest in animals with sepsis-induced lung injury. By contrast, protective MV restored PECAM1 protein levels and decreased serum levels of sPECAM1. These results imply that PECAM1 is involved in the pathogenesis of VILI and suggest that the protective mechanism of low-V_T_ plus PEEP is related to prevention or attenuation of pulmonary endothelial injury. The measurement of sPECAM1 may represent a novel, promising biomarker for monitoring the development and progression of ARDS and for the earliest identification of patients at risk of developing VILI.

## Key messages

• PECAM1 can be cleaved and shed from the surface of endothelial cells and detected as a soluble, truncated protein (sPECAM1) in the systemic circulation after a short term of injurious mechanical ventilation.

• The detection of elevated sPECAM1 serum levels during high V_T_ ventilation supports the concept of a primary endothelial cell involvement in the pathogenesis of increased vascular permeability seen in VILI and in ARDS.

• Our study suggests that low levels of sPECAM1 are indicative of normal functioning of PECAM1, whereas higher circulating levels of sPECAM1, as occurs during inflammatory conditions such as sepsis, ARDS and VILI, could serve as diagnostic and prognostic biomarkers of endothelial dysfunction.

## Abbreviations

ARDS: acute respiratory distress syndrome; CLP: cecal ligation and puncture; ELISA: enzyme-linked immunosorbent assay; MV: mechanical ventilation; PaO2/FiO2: ratio of arterial partial pressure of oxygen to fraction of inspired oxygen; PECAM1: platelet endothelial cell adhesion molecule-1; PEEP: positive end-expiratory pressure; sPECAM1: soluble platelet endothelial cell adhesion molecule-1; SpO2: oxygen saturation by pulse oxymetry; VILI: ventilator-induced lung injury; VT: tidal volume.

## Competing interests

The authors declare that they have no competing interests.

## Authors’ contributions

JV, MM, NC, CF, FV, JB and RMK conceived and designed the experiments; JV obtained funding; JV, MM, NC, FV, MLH and JLMB performed the experiments; JV, MM, NC, FV, MLH, JB and RMK coordinated data collection and data quality; JV, MM, NC, FV, CF and ML analyzed the data; JV, MM, NC, FV, MLH, CF, JB, JLMB, ML and RMK participated in the first draft of the manuscript. All authors participated in the writing process of the manuscript and read and approved the final manuscript.

## References

[B1] Pinhu L Whiteheaqd T Evans T Griffiths M Ventilator-associated lung injury Lancet 2003 361 332 340 10.1016/S0140-6736(03)12329-X 12559881

[B2] Dreyfuss D Saumon G Ventilator-induced lung injury: lessons from experimental studies Am J Respir Crit Care Med 1998 157 294 323 10.1164/ajrccm.157.1.9604014 9445314

[B3] Burns KE Adhikari NK Slutsky AS Guyatt GH Villar J Zhang H Zhou Q Cook DJ Stewart TE Meade MO Pressure and volume limited ventilation for the ventilatory management of patients with acute lung injury: a systematic review and meta-analysis PLoS One 2011 6 e14623 10.1371/journal.pone.0014623 21298026 PMC3030554

[B4] Villar J Blanco J Zhang H Slutsky AS Ventilator-induced lung injury and sepsis: two sides of the same coin? Minerva Anestesiol 2011 77 647 653 21617628

[B5] Ventilation with lower tidal volumes as compared with traditional tidal volumes for acute lung injury and the acute respiratory distress syndrome. The Acute Respiratory Distress Syndrome Network N Engl J Med 2000 342 1301 1308 10793162 10.1056/NEJM200005043421801

[B6] Villar J Herrera-Abreu MT Valladares F Muros M Pérez-Méndez L Flores C Kacmarek RM Experimental ventilator-induced lung injury Exacerbation by positive end-expiratory pressure. Anesthesiology 2009 110 1341 1347 10.1097/ALN.0b013e31819fcba9 19417614

[B7] Crosby LM Waters CM Epithelial repair mechanisms in the lung Am J Physiol Lung Cell Mol Physiol 2010 298 L715 L731 10.1152/ajplung.00361.2009 20363851 PMC2886606

[B8] Nonas SA Moreno-Vinasco L Ma SF Jacobson JR Desai AA Dudek SM Flores C Hassoun PM Sam L Ye SQ Moitra J Barnard J Grigoryev DN Lussier YA Garcia JG Use of consomic rats for genomic insights into ventilator-associated lung injury Am J Physiol Lung Cell Mol Physiol 2007 293 L292 L302 10.1152/ajplung.00481.2006 17468131 PMC3616407

[B9] Hegeman MA Hennus MP Heijnen CJ Specht PA Lachmann B Jansen NJ van Vught AJ Cobelens PM Ventilator-induced endothelial activation and inflammation in the lung and distal organs Crit Care 2009 13 R182 10.1186/cc8168 19917112 PMC2811914

[B10] Villar J Cabrera N Casula M Flores C Valladares F Muros M Blanch L Slutsky AS Kacmarek RM Mechanical ventilation modulates Toll-like receptor signaling pathway in a sepsis-induced lung injury model Intensive Care Med 2010 36 1049 1057 10.1007/s00134-010-1799-3 20397011

[B11] Villar J Cabrera NE Casula M Flores C Valladares F Diaz-Flores L Muros M Slutsky AS Kacmarek RM Mechanical ventilation modulates TLR4 and IRAK-3 in a non-infectious, ventilator-induced lung injury model Respir Res 2010 11 27 10.1186/1465-9921-11-27 20199666 PMC2841148

[B12] Woodfin A Voisin MB Nourshargh S PECAM-1: a multi-functional molecule in inflammation and vascular biology Arterioscler Thromb Vasc Biol 2007 27 2514 2523 10.1161/ATVBAHA.107.151456 17872453

[B13] Ilan N Mohsenin A Cheung L Madri JA PECAM-1 shedding during apoptosis generates a membrane-anchored truncated molecule with unique signaling characteristics FASEB J 2001 15 362 372 10.1096/fj.00-0372com 11156952

[B14] Fornasa G Groyer E Clement M Dimitrov J Compain C Gaston AT Varthaman A Khallou-Laschet J Newman DK Graff-Dubois S Nicoletti A Caligiuri G TCR stimulation drives cleavage and shedding of the ITIM receptor CD31 J Immunol 2010 184 5485 5492 10.4049/jimmunol.0902219 20400708 PMC3110943

[B15] Villar J Cabrera NE Casula M Valladares F Flores C López-Aguilar J Blanch L Zhang H Kacmarek RM Slutsky AS WNT/β-catenin signalling is modulated by mechanical ventilation in an experimental model of acute lung injury Intensive Care Med 2011 37 1201 1209 10.1007/s00134-011-2234-0 21567117

[B16] Parker JC Hernandez LA Peevy KJ Mechanisms of ventilator-induced lung injury Crit Care Med 1993 21 131 143 10.1097/00003246-199301000-00024 8420720

[B17] Tremblay LN Slutsky AS Ventilator-induced lung injury: from the bench to the bedside Intensive Care Med 2006 32 24 33 10.1007/s00134-005-2817-8 16231069

[B18] Fu Z Costello ML Tsukimoto K Prediletto R Elliott AR Mathieu-Costello O West JB High lung volume increases stress failure in pulmonary capillaries J Appl Physiol 1992 73 123 133 1506359 10.1152/jappl.1992.73.1.123

[B19] Pujin J Is the ventilator responsible for lung and systemic inflammation? Intensive Care Med 2002 28 817 819 10.1007/s00134-002-1320-8 12349817

[B20] Webb HH Tierney DF Experimental pulmonary edema due to intermittent positive pressure ventilation with high inflation pressures. Protection by positive end-expiratory pressure Am Rev Respir Dis 1974 110 556 565 4611290 10.1164/arrd.1974.110.5.556

[B21] Herrera MT Toledo C Valladares F Muros M Diaz-Flores L Flores C Villar J Positive end-expiratory pressure modulates local and systemic inflammatory responses in a sepsis-induced lung injury model Intensive Care Med 2003 29 1345 1353 10.1007/s00134-003-1756-5 12698249

[B22] Newman PJ The biology of PECAM-1 J Clin Invest 1997 100 S25 S29 10.1172/JCI119517 9413397

[B23] Newman PJ Berndt MC Gorski J White GC Lyman S Paddock C Muller WA PECAM-1 (CD31) cloning and relation to adhesion molecules of the immunoglobulin gene superfamily Science 1990 247 1219 1222 10.1126/science.1690453 1690453

[B24] Privratsky JR Paddock CM Florey O Newman DK Muller WA Newman PJ Relative contribution of PECAM1 adhesion and signaling to the maintenance of vascular integrity J Cell Sci 2011 124 1477 1485 10.1242/jcs.082271 21486942 PMC3078814

[B25] Graesser D Solowiej A Bruckner M Osterweil E Juedes A Davis S Ruddle NH Engelhardt B Madri JA Altered vascular permeability and early onset of experimental autoimmune encephalomyelitis in PECAM-1-deficient mice J Clin Invest 2002 109 383 392 10.1172/JCI0213595 11827998 PMC150854

[B26] Mahooti S Graesser D Patil S Newman P Duncan G Mak T Madri JA PECAM-1 (CD31) expression modulates bleeding time in vivo Am J Pathol 2000 157 75 81 10.1016/S0002-9440(10)64519-1 10880378 PMC1850208

[B27] Muller AM Cronen C Muller KM Kirkpatrick CJ Heterogeneous expression of cell adhesion molecules by endothelial cells in ARDS J Pathol 2002 198 270 275 10.1002/path.1186 12237888

[B28] Bhatia RK Pallister I Dent C Jones SA Topley N Enhanced neutrophil migratory activity following major blunt trauma Injury 2005 36 956 962 10.1016/j.injury.2005.03.009 15998513

[B29] Bhatt AJ Pryhuber GS Huyck H Watkins RH Metlay LA Maniscalco WM Disrupted pulmonary vasculature and decreased vascular endothelial growth factor, Flt-1, and TIE-2 in human infants dying with bronchopulmonary dysplasia Am J Respir Crit Care Med 2001 164 1971 1980 10.1164/ajrccm.164.10.2101140 11734454

[B30] Krauss T Kuhn W Lakoma C Augustin HG Circulating endothelial cell adhesion molecules as diagnostic markers for the early identification of pregnant women at risk for development of preeclampsia Am J Obst Gynecol 1997 177 443 449 10.1016/S0002-9378(97)70213-8 9290466

[B31] Soriano AO Jy W Chirinos JA Valdivia MA Velasquez HS Jimenez JJ Horstman LL Kett DH Schein RM Ahn YS Levels of endothelial and platelet microparticles and their interactions with leukocytes negatively correlate with organ dysfunction and predict mortality in severe sepsis Crit Care Med 2005 33 2540 2546 10.1097/01.CCM.0000186414.86162.03 16276178

[B32] Densmore JC Signorino PR Ou J Hatoum OA Rowe JJ Shi Y Kaul S Jones DW Sabina RE Pritchard KA Guice KS Oldham KT Endothelium-derived microparticles induce endothelial dysfunction and acute lung injury Shock 2006 26 464 471 10.1097/01.shk.0000228791.10550.36 17047516

[B33] Amabile N Heiss C Real WM Minasi P McGlothlin D Rame EJ Grossman W De Marco T Yeghiazarians Y Circulating endothelial microparticle levels predict hemodynamic severity of pulmonary hypertension Am J Respir Crit Care Med 2008 177 1268 1275 10.1164/rccm.200710-1458OC 18310479

[B34] Sinning JM Losch J Walenta K Böhm M Nickenig G Werner N Circulating CD31+/Annexin V + microparticles correlate with cardiovascular outcomes Eur Heart J 2011 32 2034 2041 10.1093/eurheartj/ehq478 21186238

[B35] Buras JA Holzmann B Sitkovsky M Animal models of sepsis: setting the stage Nat Rev Drug Discov 2005 4 854 865 10.1038/nrd1854 16224456

[B36] Goldberger A Middleton KA Oliver JA Paddock C Yan HC DeLisser HM Albelda SM Newman PJ Biosynthesis and processing of the cell adhesion molecule PECAM1 includes production of a soluble form J Biol Chem 1994 269 17183 17191 8006026

[B37] Muller WA Weigl SA Deng X Phillips DM PECAM-1 is required for transendothelial migration of leukocytes J Exp Med 1993 178 449 460 10.1084/jem.178.2.449 8340753 PMC2191108

[B38] Shehabi Y Seppelt I Pro/Con debate: is procalcitonin useful for guiding antibiotic decision making in critically ill patients? Crit Care 2008 12 211 10.1186/cc6860 18466649 PMC2481434

[B39] Chiumello D Carlesso E Cadringher P Caironi P Valenza F Polli F Tallarini F Cozzi P Cressoni M Colombo A Marini JJ Gattinoni L Lung stress and strain during mechanical ventilation for acute respiratory distress syndrome Am J Respir Crit Care Med 2008 178 346 355 10.1164/rccm.200710-1589OC 18451319

[B40] Protti A Cressoni M Santini A Langer T Mietto C Febres D Chierichetti M Coppola S Conte G Gatti S Leopardi O Masson S Lombardi L Lazzerini M Rampoldi E Cadringher P Gattinoni L Lung stress and strain during mechanical ventilation: any safe threshold? Am J Respir Crit Care Med 2011 183 1354 1362 10.1164/rccm.201010-1757OC 21297069

[B41] Slutsky AS Ranieri VM Ventilator-induced lung injury N Engl J Med 2013 369 2126 2136 10.1056/NEJMra1208707 24283226

[B42] Maas M Stapleton M Bergom C Mattson DL Newman DK Newman PJ Endothelial cell PECAM-1 confers protection against endotoxic shock Am J Physiol Heart Circ Physiol 2005 288 H159 H164 15319204 10.1152/ajpheart.00500.2004

[B43] Bhattacharya S Sen N Yiming MT Patel R Parthasarathi K Quadri S Issekutz AC Bhattacharya J High tidal volume ventilation induces proinflammatory signaling in rat lung endothelium Am J Respir Cell Mol Biol 2003 28 218 224 10.1165/rcmb.4763 12540489

[B44] Tsukimoto K Mathieu-Costello O Prediletto R Elliott AR West JB Ultrastructural appearances of pulmonary capillaries at high transmural pressures J Appl Physiol 1991 71 573 582 1718936 10.1152/jappl.1991.71.2.573

[B45] López-Aguilar J Piacentini E Villagrá A Murias G Pascotto S Saenz-Valiente A Fernández-Segoviano P Hotchkiss JR Blanch L Contributions of vascular flow and pulmonary capillary pressure to ventilator-induced lung injury Crit Care Med 2006 34 1106 1112 10.1097/01.CCM.0000205757.66971.DA 16484897

